# Androgen receptor signalling confers clonogenic and migratory advantages in urothelial cell carcinoma of the bladder

**DOI:** 10.1002/1878-0261.12957

**Published:** 2021-05-22

**Authors:** Maria V. Luna‐Velez, Jelmer J. Dijkstra, Marina A. Heuschkel, Frank P. Smit, Guillaume van de Zande, Dominique Smeets, J. P. Michiel Sedelaar, Michiel Vermeulen, Gerald W. Verhaegh, Jack A. Schalken

**Affiliations:** ^1^ Department of Urology Radboud Institute for Molecular Life Sciences Radboud University Medical Center Nijmegen the Netherlands; ^2^ Department of Molecular Biology, Faculty of Science, Radboud Institute for Molecular Life Sciences, Oncode Institute Radboud University Nijmegen the Netherlands; ^3^ MDxHealth Nijmegen the Netherlands; ^4^ Department of Genetics Radboud Institute for Molecular Life Sciences Radboud University Medical Center Nijmegen the Netherlands

**Keywords:** androgen deprivation therapy, androgen receptor, cell migration, colony formation, transcriptome analysis, urothelial cell carcinoma

## Abstract

Bladder urothelial cell carcinoma (UCC) incidence is about three times higher in men compared with women. There are several indications for the involvement of hormonal factors in the aetiology of UCC. Here, we provide evidence of androgen signalling in UCC progression. Microarray and qPCR analysis revealed that the androgen receptor (*AR*) mRNA level is upregulated in a subset of UCC cases. In an AR‐positive UCC‐derived cell line model, UM‐UC‐3‐AR, androgen treatment increased clonogenic capacity inducing the formation of big stem cell‐like holoclones, while *AR* knockdown or treatment with the AR antagonist enzalutamide abrogated this clonogenic advantage. Additionally, blockage of AR signalling reduced the cell migration potential of androgen‐stimulated UM‐UC‐3‐AR cells. These phenotypic changes were accompanied by a rewiring of the transcriptome with almost 300 genes being differentially regulated by androgens, some of which correlated with *AR* expression in UCC patients in two independent data sets. Our results demonstrate that AR signals in UCC favouring the development of an aggressive phenotype and highlights its potential as a therapeutic target for bladder cancer.

## Graphical abstract

Hormonal factors have been implicated in the aetiology of urothelial cell carcinoma (UCC). Here, we explored the role of androgen receptor (AR) signalling in UCC progression. We detected AR expression in a subset of patients with UCC. Androgen treatment increased clonogenicity and migration of a UCC‐derived cell line, while AR blockade reverted these effects. Finally, androgens regulated the expression of nearly 300 genes, some of which correlated with *AR* expression in UCC tumour tissues.

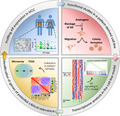

**Abbreviations**
ADT: androgen deprivation therapy
AR: androgen receptor
CIS: *in situ* carcinoma
Cp: crossing point
CSS: charcoal‐stripped serum
CYP: cytochrome P450
FDR: false discovery rate
GSEA: gene set enrichment analysis
ICC: immunocytochemistry
IHC: immunohistochemistry
MIBC: muscle‐invasive bladder cancer
NES: normalized enrichment scores
NMIBC: nonmuscle invasive bladder cancer
PC: principal component
PCA: principal component analysis
PCa: prostate cancer
PSA: prostate‐specific antigen
siRNAs: small interfering RNAs
TCGA: The Cancer Genome Atlas
UCC: bladder urothelial cell carcinoma

## 1 Introduction

Bladder urothelial cell carcinoma (UCC) is the 12th most commonly diagnosed malignancy worldwide and the 14th leading cause of cancer death among men and women [1]. UCC had an estimated 424 082 new cases in 2018 and in the same year, despite improvements in surgical techniques and perioperative care over the last decades, there were 148 270 predicted deaths due to UCC [1]. Clinically, UCC can be divided into nonmuscle invasive bladder cancer (NMIBC) and muscle‐invasive bladder cancer (MIBC), accounting for ~ 75% and 25% of the cases, respectively. NMIBC is characterized by a high recurrence rate of 30–80% in 5 years and a risk of progression to MIBC disease of 3–15% [2]. MIBC often progresses to metastatic disease, explaining the low 5‐year survival rate of about 50% after diagnosis of MIBC [3]. Identification of molecular factors involved in the development and progression of UCC is an unmet need that can help to improve diagnosis, treatment and monitoring of UCC.

Although UCC is not considered a hormone‐related type of cancer, there are indications of the involvement of hormonal factors in the aetiology of bladder cancer. The incidence of UCC in men is approximately threefold higher than in women [4, 5, 6]. For many years, this ratio was explained by the excessive exposure of men to carcinogens from cigarette smoke and industrial chemicals. However, even after correcting for these factors, the sex‐related difference persists [4]. Remarkably, this phenomenon can be reproduced in UCC animal models where carcinogen‐induced bladder tumours developed three times more often in male than in female mice [7].

Steroids are key mediators of sex‐specific differences. Androgens, the male sex steroids, exert their function through binding to the androgen receptor (AR). The AR is a transcription factor that plays a key role in the development and maintenance of male sexual organs. The androgen and AR signalling pathway is well known for its contribution to the progression of prostate cancer (PCa), and androgen deprivation therapy (ADT) is a first‐line treatment for advanced PCa [8]. Surgical castration of mice prior to carcinogen treatment also significantly reduced bladder tumour formation, and knockout of the *AR* gene completely prevented bladder carcinogenesis [9]. Interestingly, recent retrospective studies involving PCa patients that received ADT have shown that the risk of recurrence of concomitant bladder cancer was significantly reduced [10, 11]. Although the precise mechanisms by which the AR contributes to the development and progression of UCC remain poorly understood, these results highlight the importance and potential clinical relevance of elucidating the function of AR signalling in UCC.

Here, we profiled the expression of *AR* in various nonmalignant bladder and UCC specimens to correlate *AR* levels with gender and tumour stage. Next, we investigated the role of AR in several cancer‐related pathways using a new AR‐positive bladder cancer cell line model, UM‐UC‐3‐AR. Finally, we have defined the transcriptome signature accompanying phenotypical changes driven by androgen/AR signalling and used expression data from UCC patients to pin point those genes of potential clinical relevance. Our results provide new insights into the role of the AR in UCC proposing novel therapeutic routes for the treatment of this disease.

## 2 Materials and methods

### 2.1 Patient specimens

The experiments were undertaken with the understanding and written consent of each subject. The use of patient materials and the study methodologies were approved by the local ethics committee of the Radboud University Medical Center (CMO Arnhem‐Nijmegen) and conformed to the standards set by the Declaration of Helsinki. Upon transurethral resection of tumour tissue or cystectomy, specimens were snap‐frozen in liquid nitrogen. For microarray analysis, normal bladder (NB, *n* = 12), NMIBC *in situ* carcinoma (CIS, *n* = 6), Ta (*n* = 37) and T1 (*n* = 11), and MIBC T2 (*n* = 27), T3 (*n* = 14) and T4 (*n* = 13) specimens were used. For qPCR validation, independent samples were used, including NB (*n* = 5), CIS (*n* = 1), Ta (*n* = 14), T1 (*n* = 4), T2 (*n* = 7), T3 (*n* = 6) and T4 (*n* = 4). Normal bladder urothelium was obtained by macrodissection of benign urothelium from NMIBC bladder cancer specimens. Specimens were processed by step sectioning and selected for purity of benign (99–100%) or cancer cells (at least 70%). Patient and tumour characteristics can be found in Tables S1,S2.

### 2.2 RNA isolation

Total RNA was isolated using TRIzol reagent, according to the manufacturer’s instructions (Thermo Fisher Scientific, Landsmeer, the Netherlands). Concentration and purity of the RNA were determined on a NanoDrop 1000 Spectrophotometer.

### 2.3 Microarray analysis

Microarray gene expression analysis on these tissue samples was performed and described previously [12]. Normalized log_2_ values were used to determine *AR* gene expression in those specimens.

### 2.4 Real‐time semi‐quantitative RT‐qPCR

For RT‐qPCR analysis, 2 µg of DNase I‐treated total RNA was used to generate cDNA, using random hexamer primers (Roche, Almere, the Netherlands) and SuperScript II Reverse Transcriptase (Invitrogen, Landsmeer, the Netherlands). Real‐time PCR analysis was performed using LightCycler 480 SYBR Green I Master Mix and a LightCycler 480 Machine (Roche). Crossing‐point (Cp) values were determined using the LightCycler 480 SW 1.5 software (Roche). RNA not subjected to reverse transcriptase was used as a negative control. The average Cp values from two different experimental runs were used for the analysis. Expression levels of *HP1BP3* or *HPRT1* were used for normalization. Relative gene expression levels were calculated according to Pfaffl *et al*. [13]. Primers for RT‐qPCR can be found in Table S3.

### 2.5 Immunostaining

For immunohistochemistry (IHC), 4‐µm‐thick UCC tissue sections containing > 50% epithelial cells were cut. For immunocytochemistry (ICC), 5637, T24, 253J, UM‐UC‐3 and UM‐UC‐3‐AR cells (8000/well) were seeded in Lab‐Tek II 8‐Well Chamber Slides (Thermo Fisher). For AR nuclear translocation experiments, cells were seeded in androgen‐deprived medium. After 24 h, cells were treated for 24 h with 10 nm R1881 (Organon, Oss, the Netherlands) or EtOH (0.1%) as vehicle control. Cells were fixed with 4% paraformaldehyde and left to dry overnight. Slides with fixed tissue (IHC) or cells (ICC) were exposed to boiling 10 mm sodium citrate (pH 6.0) for 10 min. The rabbit polyclonal AR antibody N20 (Santa Cruz, SC‐816, Heidelberg, Germany) was diluted 1 : 5000 in PBS/1% BSA and incubated for 1 h at room temperature, followed by exposure to Poly‐HRP goat anti‐Ms/Rb/Rt IgG (DPVO110HRP, Immunologic) solution diluted 1 : 1 in PBS/1% BSA for 30 min. Slides were incubated with Bright‐DAB (Immunologic) for 10 min, counterstained with haematoxylin and mounted in Permount Mounting Medium (Thermo Fisher Scientific).

### 2.6 Cell culture

The UM‐UC‐3 cell line was purchased from the ATCC (CRL‐1749, Molsheim Cedex, France). The fully AR‐positive UM‐UC‐3‐AR line was obtained from the UM‐UC‐3 parental line by clonal selection. UM‐UC‐3 and UM‐UC‐3‐AR cells were cultured in Dulbecco’s modified Eagle medium with 1 g·L^−1^ d‐glucose, 4 mm l‐glutamine and 1 mm pyruvate, supplemented with 10% FCS (Sigma) and 1x nonessential amino acids (Gibco, Landsmeer, the Netherlands). The 5637 (HTB‐9), T24 (HTB‐4), 253J [14] and LNCaP (CRL‐1740) cell lines were cultured in Roswell Park Memorial Institute 1640 Medium, supplemented with 2 mm l‐glutamine and 10% FCS. All cell cultures were maintained in a humidified atmosphere at 37 °C and 5% CO_2_. Cells were frequently tested for *Mycoplasma* infection, using a *Mycoplasma*‐specific PCR. All cell lines were authenticated using the PowerPlex 21 System (Promega, Leiden, the Netherlands) by Eurofins Genomics (Germany) and propagated for no more than 6 months or 30 passages after resuscitation from the authenticated stocks.

### 2.7 Western blot analysis

A total of 1.2 × 10^6^ of UM‐UC‐3, UM‐UC‐3‐AR, LNCaP and 5637 cells were seeded per well in 6‐well plates. Twenty four hours later, cells were lysed using Laemmli lysis buffer (1 mm CaCl_2_, 2% SDS, 60 mm Tris/glycine pH 6.8) supplemented with 300 mm β‐mercaptoethanol. Lysates were homogenized by sheering them through a 0.5 × 25‐mm syringe needle. The protein concentration was measured after staining with Coomassie Brilliant Blue (Merck, Amsterdam, the Netherlands) using BSA as a standard. A total of 50 µg of protein was used for SDS/PAGE using 7.5% polyacrylamide gels. Proteins were electrotransferred onto PVDF membranes (Hybond 0.45 µm; Amersham Biosciences, Buckinghamshire, UK). Membranes were blocked for 1 h in PBS‐T/5% nonfat dry milk and incubated overnight with the primary antibody. The rabbit polyclonal AR antibody N20 (Santa Cruz, SC‐816) and the mouse monoclonal antibody anti‐β‐actin (Sigma‐Aldrich; clone AC‐15) were diluted 1 : 50 000 and 1 : 5000 in PBS‐T/5% nonfat dry milk, respectively. The PO‐conjugated donkey anti‐rabbit antibody (Amersham Biosciences, N4934) or sheep anti‐mouse antibody (Amersham Biosciences, NXA931) diluted 1 :50 000 in PBS‐T was used as secondary antibodies. Protein bands were detected using the ECL Detection Kit and Hyperfilm (Amersham Biosciences).

### 2.8 Karyotyping

UM‐UC‐3 and UM‐UC‐3‐AR were seeded in T25 culture flasks until reaching 40% confluency. Cells were arrested in metaphase by exposing them to 0.2 µg·mL^−1^ of KaryoMAX (Life Technologies, Bleijswijk, the Netherlands) for 30 min. From each cell line, five metaphases were fully analysed by GTG banding at ~ 450 band level to exclude gross chromosomal aberrations. Count and analysis of chromosome marks were manually executed using reference marks described for UM‐UC‐3 by the ATCC: m1 = der(1)t(1;?) (p32;?), m2 = ?t(1p5p), m3 = i(3q), m4 = t(7q14q) and m5 = ?t(2p3p).

### 2.9 Glycine repeat and gene copy number

Genomic DNA from UM‐UC‐3 and UM‐UC‐3‐AR was isolated using the QIAaMP DNA Mini Kit (Qiagen, Venlo, the Netherlands) following the manufacturer’s instructions. For amplification of the *AR* gene glycine repeat region, Phusion High‐Fidelity Polymerase and the Phusion High‐Fidelity buffer specialized for GC‐rich regions (Thermo Fisher) and primers listed in Table S4 were used. The purified DNA was subjected to Sanger DNA sequencing. Gene copy number was determined by qPCR analysis using genomic DNA as a template and primers against *AR, SPIN4, PCA3* and *GAPDH* intronic regions. *PCA3* and *GAPDH* were used for normalization. Normalized *AR* and *SPIN4* copy numbers were adjusted to those from healthy female genomic DNA. Copy number of both genes from a benign prostate hyperplasia specimen was used as a control.

### 2.10 KLK3 luciferase reporter assay

A total of 10 000 cells/well were seeded in FCS‐containing medium in 96‐well plates in triplicate. Transfection mix containing 90 ng of the pCMV‐PSA85‐Luc Reporter Construct (kindly provided by J. Trapman; Erasmus MC, Rotterdam), 10 ng of the pRL‐TK *Renilla* (Promega) control vector, and X‐tremeGENE™ 9 Transfection Reagent (Roche) was added per well. One day after transfection, 0.1 or 0.5 nm of R1881 (Organon) was added to the medium in combination with 1 or 5 µm enzalutamide (Selleck Chemicals, Houston, TX, USA), respectively. DMSO and EtOH (< 1%) were used as vehicle controls. The luciferase activities were measured 16 h later using the Dual‐Luciferase Reporter Assay System (Promega). Luciferase signals were measured on a VVICTOR3 Multilabel Reader (Perkin Elmer, Groningen, the Netherlands).

### 2.11 Small interfering RNA Transfection

Silencer Select small interfering RNAs (siRNAs) directed against the *AR* and a negative control No. 1 siRNA were used (Ambion, Bleijswijk, the Netherlands; Table S5). Cells were transfected with siRNAs at a 20 nm final concentration using Lipofectamine RNAiMAX Transfection Reagent according to the manufacturer’s protocol (Thermo Fisher Scientific).

### 2.12 Colony formation assay

UM‐UC‐3 and UM‐UC‐3‐AR cells were seeded in 6‐well plates at a density of 250 cells/well and cultured in medium containing 0.1 nm R1881 (Organon) in combination with 1 µm enzalutamide (Selleck Chemicals). DMSO and EtOH (0.2%) were used as vehicle controls. For siRNA experiments, 200 000 cells were seeded and transfected as described above. One day post‐transfection, cells were harvested and reseeded in 6‐well plates in the presence of 0.1 nm R1881. Medium was refreshed after 1 week. After 14 days, colonies were washed with PBS, fixed with 3% paraformaldehyde, and stained with 0.01% crystal violet (Merck) for 60 min. The number and size of colonies were analysed using the fiji software [15]. Morphological analysis of colonies was done using light microscopy.

### 2.13 Cell proliferation assay

A total of 10 000 cells/well were seeded in 96‐well plates in triplicate, in charcoal‐stripped serum (CSS)‐containing medium to wash away traces of androgens previously reported to be present in FCS [16]. One day after seeding, 0.1 nm of synthetic androgen R1881 (Organon) was added to the medium in combination with 1 µm enzalutamide (Selleck Chemicals). DMSO and EtOH (0.2%) were used as vehicle controls. For siRNA experiments, 10 000 cells/well were seeded and transfected as described above. One day after transfection, cells were treated with R1881 and enzalutamide at concentrations described before. Cell viability was measured at regular time intervals using 3‐(4,5‐dimethylthiazol‐2‐yl)‐2,5‐diphenyltetrazolium bromide (1 mg·mL^−1^) assays. Doubling time was calculated using the Doubling Time tool at www.doubling‐time.com.

### 2.14 Cell migration assay

A total of 70 000 UM‐UC‐3 or UM‐UC‐3‐AR cells were seeded per well in a 24‐well plate CSS‐containing medium. One day after seeding, cells were treated with 0.1 nm R1881 (Organon) in combination with 1 µm enzalutamide (Selleck Chemicals). DMSO and EtOH (0.2%) were used as vehicle controls. For siRNA‐mediated *AR* knockdown, siRNA transfection was performed 24 h after seeding. Treatment with 0.1 nm R1881 started 24 h after transfection. When cells reached 100% confluency (72 h after hormonal manipulation), a wound was made by mechanically scratching the cell monolayer with a 1‐mL pipette tip. Medium was replaced, and cells were allowed to migrate into the cell‐free area. Wound size was measured at regular time intervals, and cell migration was calculated using the difference in wound size between each time point.

### 2.15 RNA sequencing sample preparation

For RNA sequencing analysis, samples were purified using an RNeasy RNA Extraction Kit (Qiagen) with DNase I treatment. Total RNA (1 µg per sample) was used for library preparation using the RNA HyperPrep Kit with RiboErase (KAPA Biosystems, Almere, the Netherlands). A fragmentation step was carried out for 6.5 min, and NEXTFLEX DNA barcodes (Sanbio, Uden, the Netherlands) were used for adapter ligation. Libraries were amplified using 10 amplification cycles. Library concentration was measured using the dsDNA Fluorescence Quantification Assays (DeNovix, Wilmington, Delaware, the Netherlands), and library size was determined using the BioAnalyzer High Sensitivity DNA Kit (Agilent, Amstelveen, the Netherlands). Sequencing was performed using an Illumina NextSeq 500, and 50‐bp paired‐end reads were generated.

### 2.16 RNA sequencing analysis

Paired‐end reads were aligned to the human GRCh38 genome (Release 95—January 2019) using the STAR RNA‐seq aligner version 2.4.0 [17], with –sjdbOverhang set to 100. Mapped reads were counted with HTSeq count [18] with the following settings: ‐r pos –s reverse –t exon. Differential gene expression was determined with DESeq2 [19], and genes with a false discovery rate (FDR) < 0.05 were considered to be differentially expressed. Visualization was performed using the *Complex Heatmap* package [20].

### 2.17 Gene set enrichment analysis

Gene set enrichment analysis (GSEA) was done using the GSEA software supplied by the Broad Institute [21]. The gene lists from R1881 (Organon)/enzalutamide (Selleck Chemicals) or siControl/siAR were ranked based on the fold change after shrinkage using DESeq2 [19]. The *Hallmarks of Cancer* gene set database was used with permutations set to 1000 to determine the FDR. Normalized enrichment scores (NESs) were obtained after analysis.

### 2.18 TCGA data

Expression data (FPKM values) of *AR* and of androgen‐responsive genes identified in UM‐UC‐3‐AR are available from the public The Cancer Genome Atlas (TCGA) database, containing 408 MIBC cases (Tables S6,S7). The clinical and expression data were downloaded from cBioPortal [22].

### 2.19 Statistical analyses

The data of bar and dot plots are presented as means ± SEM from at least three independent experiments. Two‐tailed paired and unpaired *t*‐tests were performed using graphpad prism (GraphPad Software, Inc., San Diego, CA, USA). A *P*‐value of < 0.05 was considered statistically significant, and *P* < 0.05 is represented by one star (*), *P* < 0.01 is represented by two stars (**), *P* < 0.001 is represented by three stars (***) and *P* < 0.0001 is represented by four stars (****).

For correlations of TCGA data, FPKM values were log_2_‐transformed (values equal to 0 were added +1 before transformation). Spearman’s correlation of log_2_‐transformed expression values (TCGA or microarray) was calculated using the R package *psych* [23]. A correlation was considered statistically significant when having an FDR < 0.05 and valid when the correlation coefficient was higher than 0.3 or lower than −0.3 [24]. The built‐in R function *prcomp* from the package *stats* [25] was used to perform the principal component analysis (PCA) and *ggplot2* from the package *tydiverse* [26] was used for visualization of results.

## 3 Results

### 3.1 AR is upregulated in a subset of UCC cases

**Fig. 1:**
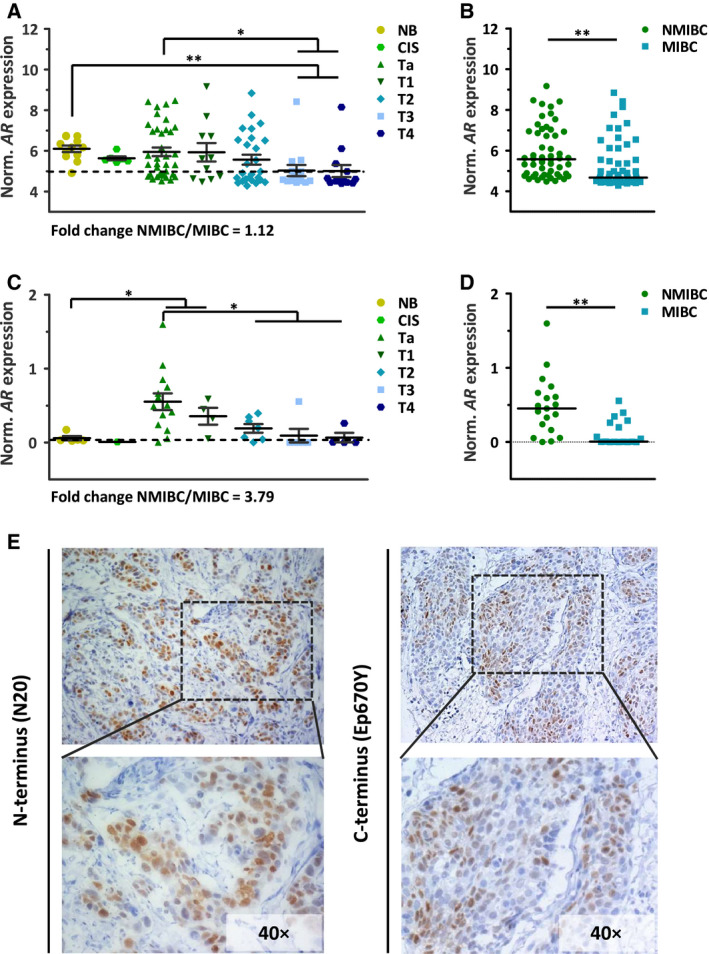
AR expression in urothelial bladder (cancer) tissue samples. (A) Microarray analysis showing *AR* gene expression profiles in nonmalignant bladder (NB, *n* = 12), NMIBC *in situ* (CIS, *n* = 6), Ta (*n* = 37) and T1 (*n* = 11), and MIBC T2 (*n* = 27), T3 (*n* = 14) and T4 (*n* = 13). Microarray values (log_2_) shown as the mean ± SEM of each group are depicted. Unpaired *t*‐test; *, *P* < 0.05; **, *P* < 0.01. Horizontal dotted line marks the median = 5 and separates high‐AR from low‐*AR* tumour samples. A subset of UCC tissue samples (27/108) express *AR* mRNA at levels above upper limit from the 95% confidence interval (CI) of *AR* expression in nonmalignant tissue. (B) Median *AR* expression values (log_2_) from microarray data in NMIBC (*n* = 54) and MIBC (*n* = 54). Unpaired *t*‐test; **, *P* < 0.01. (C) *AR* mRNA expression obtained by RT‐qPCR and normalized to that of *HP1BP3* in nonmalignant bladder (NB, *n* = 5) and in CIS (*n* = 1), Ta (*n* = 14), T1 (*n* = 4), T2 (*n* = 7), T3 (*n* = 6) and T4 (*n* = 4) tumour specimen. Horizontal dotted line marks the median = 0.26 and separates high‐ from low‐*AR* samples. A subset of UCC tissue samples (21/36) express *AR* mRNA at levels above upper limit from the 95% CI of *AR* expression in nonmalignant tissue. Unpaired *t*‐test; **, *P* < 0.01. (D) Median *AR* expression values in NMIBC (*n* = 19) and MIBC (*n* = 17). Unpaired *t*‐test; *, *P* < 0.05. (E) Immunohistochemical analysis of an UCC T3 tumour stained with the AR N20 antibody (N terminus‐specific) and Ep670Y antibody (C terminus‐specific). Haematoxylin was used as nuclear counterstain. The dotted rectangular area is 40× magnified and the upper images 20x.

To profile the expression of *AR* mRNA in urothelial bladder tissue, we performed a microarray analysis on NMIBC (CIS, and tumours of Ta and T1 stage), MIBC (tumours of T2, T3 and T4 stages) and nonmalignant bladder urothelial specimens. A subset of UCC tissue samples (27/108) expressed *AR* mRNA at levels above those from nonmalignant tissue (Fig. 1A). *AR* mRNA expression was significantly higher in NMIBC compared with MIBC tissues (Fig. 1B), and a trend towards lower AR expression and tumour stage was observed. The number of male UCC cases was about three times higher than female cases (Fig. S1A), but no significant difference in *AR* expression was observed between the two groups (Fig. S1B). A technical validation by RT‐qPCR, including additional UCC cases, confirmed the association between *AR* expression and low stage UCC observed in the microarray data (Table S8). More than 55% of the UCC cases (21/36) have *AR* mRNA levels above nonmalignant tissue, and 70% of those were NMIBC cases (Fig. 1C,D). Also in the validation cohort, no significant difference in *AR* mRNA levels was observed between male and female cases (Fig. S1C,D). To assess AR protein expression and localization, we performed IHC analysis on tumour sections from the MIBC (T3) specimen with the highest *AR* mRNA expression from our microarray cohort, using the AR N and C terminus‐specific antibodies, N20 and Ep670Y, respectively. A heterogeneous population of AR‐positive and AR‐negative tumour cells was observed. In the AR‐positive cells, the AR was located in the nucleus. Androgen stimulation promotes AR nuclear translocation, a prerequisite for AR‐mediated transcriptional activity, and hence suggests active AR signalling (Fig. 1E).

### 3.2 UM‐UC‐3‐AR as a new AR‐positive UCC‐derived cell line model

**Fig. 2:**
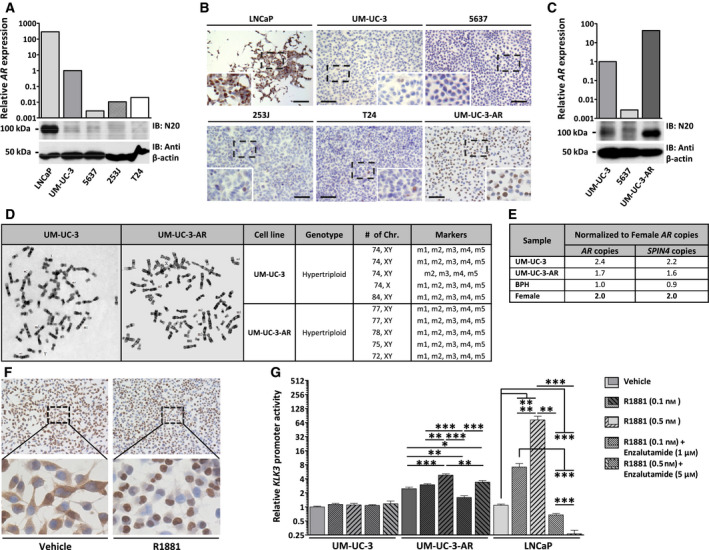
AR expression and signalling in UM‐UC‐3‐AR. (A and C) Relative *AR* mRNA expression levels determined by RT‐qPCR and normalized to that of *HP1BP3* in UCC‐derived cell lines 253J, 5637, T24, UM‐UC‐3 and UM‐UC‐3‐AR and in LNCaP PCa cells. Western blot analysis of AR protein levels detected by AR N terminus antibody N20. Protein levels of β‐actin (anti‐β‐actin) were used as a protein loading control. (B) ICC analysis of AR in LNCaP, 253J, 5637, T24, UM‐UC‐3 and UM‐UC‐3‐AR cells grown in FCS‐containing medium. Cells were stained with the N20 antibody, and haematoxylin was used as nuclear counterstain. Scale bar represents 100 µm. (D) Karyotyping analysis of UM‐UC‐3 and UM‐UC‐3‐AR cells arrested in metaphase. Genotype, number of chromosomes and chromosomal markers are depicted. (E) *AR* and *SPIN4* gene copy numbers determined by qPCR using genomic DNA from UM‐UC‐3 and UM‐UC‐3‐AR cells, benign prostate hyperplasia (BPH) and white blood cells from a healthy female. PCR values of the X‐linked genes were normalized to PCR values of the autosomal *PCA3* and *GAPDH* genes, and then normalized to the ratio found in female cells. (F) Cellular localization of the AR protein in UM‐UC‐3‐AR cells grown in androgen‐depleted medium and treated with 10 nm R1881 or vehicle control. Cells were stained with the N20 antibody (brown), and haematoxylin was used as nuclear counterstain. (G) *KLK3* reporter activity in UM‐UC‐3, UM‐UC‐3‐AR and LNCaP cells. Cells were transfected with the PSA85‐Luc firefly luciferase reporter vector and the pRL‐TK *Renilla* luciferase control vector. Cells were grown in FCS‐containing medium and treated with R1881 in combination with enzalutamide or vehicle control. Bars represent the mean ± SEM of three independent experiments performed in triplicate. Unpaired *t*‐test; *, *P* < 0.05; **, *P* < 0.01; ***, *P* < 0.001.

For functional experiments, we assessed AR expression in the previously described AR‐positive bladder carcinoma‐derived cell line models 253J, T24 and UM‐UC‐3 [27, 28]. All three cell lines expressed *AR* at low levels, several orders of magnitude lower than the PCa cell line LNCaP. Of the three UCC cell lines, UM‐UC‐3 showed the highest *AR* expression (Fig. 2A). At the protein level, all three cell lines showed very weak AR protein expression levels, almost indistinguishable from AR‐negative 5637 cells (Fig. 2A). ICC analysis revealed that 253J, T24 and UM‐UC‐3 cells are very heterogeneous populations with roughly 3% of cells being AR‐positive (Fig. 2B). UM‐UC‐3 cells were then subcloned using limited dilution, in order to obtain a fully AR‐positive cell line (UM‐UC‐3‐AR). UM‐UC‐3‐AR expressed AR in more than 90% of the cells (Fig. 2B), and this percentage remained constant for over 40 passages (data not shown). At the mRNA level, expression of *AR* was 40 times higher in UM‐UC‐3‐AR than in the parental line UM‐UC‐3 (Fig. 2C). These *AR* levels were comparable to those in *AR*‐positive UCC tissue and nonmalignant bladder urothelium, respectively (Fig. S2A). *AR* mRNA expression in UM‐UC‐3 and UM‐UC‐3‐AR was consistent with the difference observed at protein levels (Fig. 2C). Using five chromosome markers described for parental UM‐UC‐3, karyotyping analysis confirmed that the UM‐UC‐3‐AR clone is derived from parental UM‐UC‐3 (Fig. 2D). These data indicate that UM‐UC‐3‐AR is an UM‐UC‐3‐derived AR‐positive UCC cell line model.

To determine whether the elevated *AR* mRNA levels in UM‐UC‐3‐AR were a consequence of *AR* gene amplification, qPCR of genomic DNA of UM‐UC‐3 and UM‐UC‐3‐AR was performed. Both cell lines carried two copies of the *AR* gene, resulting from a duplication of the X chromosome (Fig. 2E). The *AR* gene contains two repeat regions within the N‐terminal domain: a glutamine (CAG) and a glycine (GGC) tract. More than 16 glycine (GGC) copies have been demonstrated to form a hairpin structure that inhibits translation [29]. Both UM‐UC‐3 and UM‐UC‐3‐AR carried 17 glycine repeats in exon 1 of the *AR* gene (Fig. S2B). Although these results do not explain the AR expression difference between the two cell lines, they further underscore that UM‐UC‐3‐AR is derived from UM‐UC‐3.

Nuclear staining of AR in UM‐UC‐3‐AR cells (Fig. 2B) could be due to androgens present in the FCS‐containing medium that was used for these experiments. The androgen levels present in FCS are sufficient to promote AR activation in PCa cell lines [16]. To show that AR nuclear translocation in UM‐UC‐3‐AR is indeed a response to androgen stimulation, cells were cultured in CSS‐containing media and media containing the synthetic androgen R1881. In androgen‐depleted medium (CSS), the AR protein was found in the cytoplasm of UM‐UC‐3‐AR cells, whereas androgen stimulation promoted its translocation into the nucleus (Fig. 2F). A similar response was observed in parental UM‐UC‐3 cells (Fig. S2C).

Sequencing analysis did not identify mutations in the *AR* gene from UM‐UC‐3‐AR (data not shown), indicating that it should encode for a functional protein. As a transcription factor, the main role of AR is to promote transcriptional activation of AR‐targeted genes. The *KLK3* gene, encoding the prostate‐specific antigen (PSA), is a well‐established AR‐responsive gene [30]. By using a *KLK3 luciferase* reporter construct, we were able to monitor AR‐mediated transcription in UM‐UC‐3, UM‐UC‐3‐AR and LNCaP cells. In a dose‐dependent manner, androgen stimulation significantly increased AR‐mediated transcriptional activity observed in FCS‐containing media in UM‐UC‐3‐AR and LNCaP, but not in UM‐UC‐3 cells (Fig. 2G). Treatment with enzalutamide, a new‐generation AR antagonist, at on‐target doses [31, 32], significantly decreased transcriptional activity of AR in UM‐UC‐3‐AR and LNCaP, but not in parental UM‐UC‐3 cells (Fig. 2G). Altogether, these results indicate that UM‐UC‐3‐AR cells express a functional AR.

### 3.3 AR signalling induces colony formation in UCC‐derived cell line

**Fig. 3:**
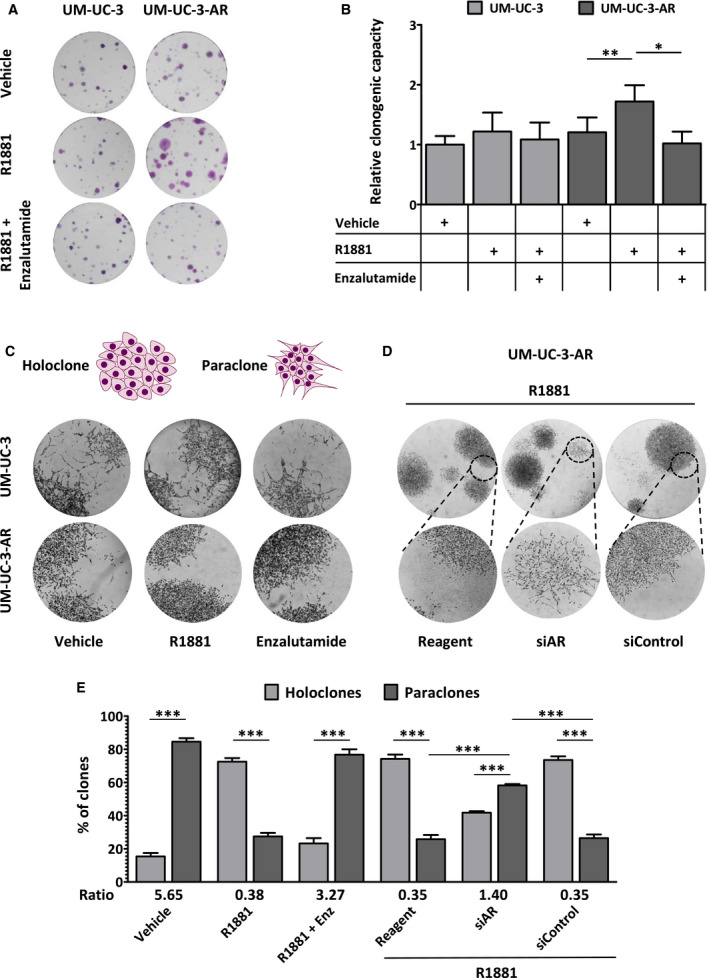
Androgen/AR‐dependent UM‐UC‐3‐AR colony formation. (A) Representative images of colony‐forming assays of UM‐UC‐3 and UM‐UC‐3‐AR cells grown in androgen‐depleted medium and treated with 0.1 nm R1881 with or without 1 µm enzalutamide, compared with vehicle control conditions. (B) Relative clonogenic capacity of UM‐UC‐3 and UM‐UC‐3‐AR cells that were treated with 0.1 nm R1881 in combination with 1 µm enzalutamide or vehicle control. Bars represent the mean ± SEM of three independent experiments performed in triplicate. Paired *t*‐test; *, *P* < 0.05; **, *P* < 0.01. (C) Illustration of holoclone and paraclone morphology. Representative light microscopy images of UM‐UC‐3 and UM‐UC‐3‐AR colonies after hormonal manipulation. (D) Light microscopic images of UM‐UC‐3‐AR colonies that were grown in R1881 medium following *AR* knockdown. Upper images, 20× magnification; dotted circles mark regions visualized at 40x magnification (lower images). (E) Percentage of holoclone and paraclone colonies formed by UM‐UC‐3‐AR cells following hormonal manipulation (shown in C) or after siRNA‐mediated *AR* knockdown (shown in D). Bars represent the mean ± SEM of three independent experiments performed in triplicate. Unpaired *t*‐test; ***, *P* < 0.001.

Androgens stimulate cell proliferation in PCa cells [33]. Therefore, we assessed whether androgens could promote proliferation of the UCC‐derived UM‐UC‐3‐AR cell line. Treatment with R1881 reduced doubling time of LNCaP cells, whereas the addition of enzalutamide reverted the effect. Neither treatment, however, had an effect on UM‐UC‐3 or UM‐UC‐3‐AR cell proliferation (Fig. S3A). Likewise, siRNA‐mediated knockdown of *AR* resulted in a significant increase in the doubling time of hormone‐stimulated LNCaP cells, but had no effect on UM‐UC‐3‐AR cells (Fig. S3B) despite an *AR* knockdown of about 80% and 50% at the RNA and protein levels, resp. (Fig. S3C,D). Remarkably, androgen treatment increased the clonogenic capacity of UM‐UC‐3‐AR, but not of parental UM‐UC‐3 cells, and addition of enzalutamide abrogated this clonogenic advantage (Fig. 3A,B). Based on their morphology and growth potential, individual clones can be classified into holoclones (*holo* = entire), paraclones (*para* = beyond) or meroclones (*mero* = partial) [34, 35]. Morphological analysis of the colonies showed that UM‐UC‐3 colonies had irregular edges, and scattered and flattened cells. These so‐called paraclones were also observed with UM‐UC‐3‐AR colonies in androgen‐depleted conditions (Fig. 3C). In contrast, androgen stimulation of UM‐UC‐3‐AR induced the formation of tightly packed, round holoclones with better defined borders (Fig. 3C). To our surprise, although *AR* knockdown did not achieve a significant effect in the overall colony formation (Fig. S3E,F), it did significantly increase the number of UM‐UC‐3‐AR paraclones despite hormone stimulation (Fig. 3D,E). These data indicate that the morphologic changes observed in UM‐UC‐3‐AR were dependent on both AR and androgens.

### 3.4 AR increases cell migration capacity of UM‐UC‐3‐AR

**Fig. 4:**
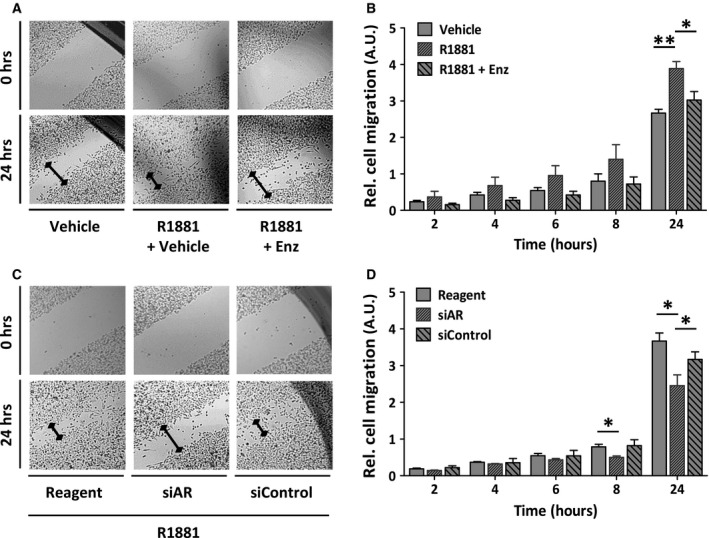
Effect of AR signalling in the migration capacity of UM‐UC‐3‐AR. (A) Migration of UM‐UC‐3‐AR cells as determined by scratch assays. Cells were grown in androgen‐depleted media and treated with 0.1 nm R1881 in combination with 1 µm enzalutamide (Enz) or vehicle control. Light microscopic images taken at 0 and 24 h after scratching (40× magnification). (B) Relative cell migration of UM‐UC‐3‐AR cells as determined by scratch assays. Decrease in wound size at individual time points compared to t = 0 was measured and used to calculate the migration capacity of cells. Unpaired t‐test; *, *P* < 0.05; **, *P* < 0.01. (C) Migration of UM‐UC‐3‐AR cells 96 h after siRNA‐mediated *AR* knockdown in medium containing 0.1 nm R1881. Light microscopic images taken at 0 and 24 h after scratching. (D) Relative cell migration of UM‐UC‐3‐AR cells following siRNA‐mediated *AR* knockdown. Unpaired *t*‐test; *, *P* < 0.05. Bars represent the mean ± SEM of three independent experiments performed in triplicate.

Among the three types of colonies, holoclones have the greatest growth potential [34]. The ability of UM‐UC‐3‐AR cells to form big colonies with a holoclone‐like morphology in response to androgen stimulation proposes a role of AR signalling in the acquisition of a more aggressive phenotype. In addition to the high clonogenic capacity of aggressive tumour cells, these cells often gain cell migratory and invasive properties. Therefore, we evaluated the effect of AR signalling on UM‐UC‐3‐AR cell migration using a scratch assay. Indeed, hormone stimulation increased migration of UM‐UC‐3‐AR cells and this effect was reverted upon treatment with enzalutamide (Fig. 4A,B) or upon siRNA‐mediated knockdown of *AR* expression (Fig. 4C,D). Cell migration of parental UM‐UC‐3 cells was not affected by androgen and *AR* modulation (Fig. S4).

### 3.5 Androgen/AR signalling in UM‐UC‐3‐AR

**Fig. 5:**
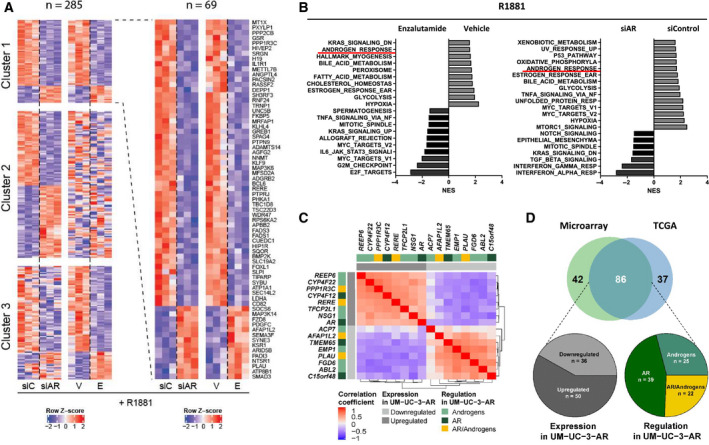
Transcriptome dynamics in UM‐UC‐3‐AR following androgen stimulation or *AR* knockdown. (A) Two row‐matched heat maps (left) and a detailed heat map of cluster 1 (right). Rows show *Z*‐scores of mRNA abundance from significantly changing genes (FDR < 0.05). UM‐UC‐3‐AR cells were stimulated with 0.1 nm R1881 or DMSO vehicle control (V) in combination with 1 µm enzalutamide (E). Cluster 1 shows genes that are differentially expressed in both comparisons; cluster 2 shows genes that are only significantly changing after knockdown of AR; cluster 3 shows genes that are only significantly changing after androgen stimulation. (B) GSEA revealing pathways significantly enriched in R1881/enzalutamide (Enz) and siControl/siAR conditions (FDR < 0.01). Androgen response is underlined (red) as a positive control. NESs are shown. (C) Genes significantly correlating with *AR* mRNA expression (FDR < 0.05) with a correlation coefficient of > 0.3 or < −0.3 in our UCC microarray data set. Matrix shows correlation coefficients from each intersection. A description of the type of regulation observed for each gene in UM‐UC‐3‐AR is depicted. (D) Venn diagram displaying the number of genes that showed expression dynamics similar to those observed in UM‐UC‐3‐AR, in our microarray and in the TCGA data sets. Number in the intersection represents those genes in common between both data sets, and lower circles describe the type of regulation observed in UM‐UC‐3‐AR for this group of genes.

Active AR signalling should impact transcriptional output. To study the androgen/AR‐driven gene expression dynamics in the UM‐UC‐3‐AR, we analysed transcriptome data obtained by RNA sequencing of hormone‐stimulated cells and compared this to the gene expression profile obtained after enzalutamide treatment or *AR* knockdown. Clustering analysis revealed 285 differentially expressed genes (FDR < 0.05) (Fig. 5A, Table S9). A total of 163 genes displayed an androgen‐responsive pattern (cluster 1 and 3, Fig. 5A), with 69 of them being dependent on both androgen and AR signalling (cluster 1, Fig. 5A). Among these genes were *FKBP5* and *GREB1,* known AR‐targeted genes. Curiously, a subset of genes were significantly downregulated upon *AR* knockdown but not by enzalutamide treatment (cluster 2, Fig. 5A), possibly indicating nongenomic (i.e. non‐nuclear) AR signalling, which has been previously described in PCa [35]. To disentangle gene expression signatures from our transcriptome analysis, GSEA was performed. Transcripts of genes involved in hypoxia, glycolysis, bile acid metabolism, oestrogen response and androgen response constituted the gene expression signature associated with androgen and AR signalling in our UCC cell line model (Fig. 5B). Collectively, these results confirm an active androgen/AR signalling in UM‐UC‐3‐AR cells.

We next assessed the expression levels of all androgen‐responsive genes that were identified in UM‐UC‐3‐AR, in our UCC cohort. Expression values of *AR* and 265 out of 284 genes were present in our microarray data set (Table S10). First, we separated our microarray UCC cases into high‐*AR* (*n* = 48; NMIBC = 30 and MIBC = 18) and low‐*AR* (*n* = 49; NMIBC = 18 and MIBC = 31), using median *AR* expression as a cut‐off. We then compared the median expression levels of the androgen‐responsive genes between these two groups. A total of 128/265 genes from our UCC cohort displayed an expression pattern we defined as consistent with the regulation observed in UM‐UC‐3‐AR. In brief, from the genes downregulated by enzalutamide treatment and/or upon siRNA‐mediated *AR* knockdown in UM‐UC‐3‐AR 75/175 showed similar expression dynamics to *AR* in the high‐*AR* and low‐*AR* groups. From the genes upregulated after enzalutamide treatment and/or *AR* knockdown in our cell line model, the expression of 53/90 genes was higher in the low‐*AR* group compared with the high‐*AR* group (Table S11). A Spearman correlation test between *AR* and these 128 genes revealed two gene clusters (Fig. S5A), one composed of genes negatively correlating with *AR* (cluster 1) and one of genes that positively correlated with *AR* (cluster 2). From the genes, which expression significantly (FDR < 0.05) correlated with *AR* levels, 15 genes had a correlation coefficient > 0.3 or < −0.3 (Fig. 5C, Table S12). In an independent cohort of 408 MIBC cases, from the TCGA database, 123/254 genes were consistent with the regulation observed in UM‐UC‐3‐AR (Table S13) and 86 of these were among those previously identified in our microarray cohort (Fig. 5D). Four genes, namely *PPP1R3C, CYP4F12, RERE* and *PLAU,* had a correlation coefficient > 0.3 or < −0.3 in both data sets (Table S14). As expected, *PPP1R3C, CYP4F12* and *RERE* were significantly upregulated in the high‐*AR* compared with the low‐*AR* group from both cohorts, while *PLAU*, was significantly downregulated in the high‐ vs low‐*AR* cases in both data sets (Fig. S5B). These results propose a role for AR in the regulation of these four genes in UCC.

### 3.6 Influence of subclass and gender in androgen/AR regulation in UCC

**Fig. 6:**
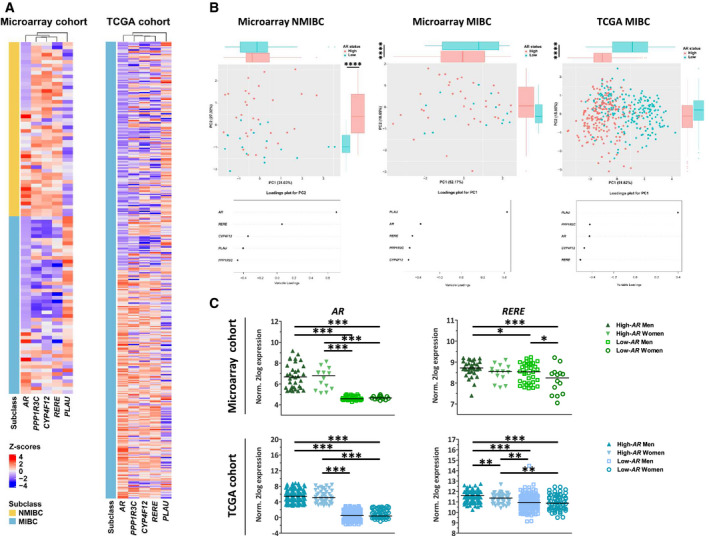
Influence of subclass and gender in the androgen/AR signalling in UCC. (A) Row‐matched heat maps of expression values of *AR, PPP1R3C, CYP4F12, RERE* and *PLAU* from our microarray NMIBC (left) and MIBC (centre) cases and from MIBC (right) from TCGA data. Columns show *Z*‐scores of log_2_ expression values. Rows correspond to individual patients. (B) Principal component analysis (PCA) of microarray NMIBC (left), MIBC (center) and MIBC from TCGA (right) cases separated based on expression of *AR, PPP1R3C, CYP4F12, RERE* and *PLAU*. Loadings for each PCA are depicted below. (C) Median *AR* and *RERE* expression values (log_2_) from microarray UCC cases in high‐*AR* men (*n* = 34), high‐*AR* women (*n* = 14), low‐*AR* men (*n* = 35) and low‐*AR* women (*n* = 14), and from TCGA data in high‐*AR* men (*n* = 157), high‐*AR* women (*n* = 47), low‐*AR* men (*n* = 144) and low‐*AR* women (*n* = 60). Unpaired t‐test; *, *P* < 0.05; **, *P* < 0.01; and ***, *P* < 0.001.

An expression heat map of *AR, PPP1R3C, CYP4F12, RERE* and *PLAU* revealed that the correlation of expression of *AR* and *PPP1R3C, CYP4F12*, *RERE* and *PLAU* was stronger in MIBC compared with NMIBC tissue Fig. 6A). Indeed, clustering analysis showed that separation of patients based on expression of *PPP1R3C, CYP4F12, RERE* and *PLAU* clearly separated the high‐ and low‐*AR* groups in the TCGA cohort (Fig. 6A). To further verify these findings, we performed a PCA. The first two principal components (PCs) in NMIBC described 58.35% of the variance in the data and PC2, which explained 27.32% of the data, was driven by *AR* expression (Fig. 6B). Interestingly, analysis of the PC loadings showed that in PC2 *PPP1R3C, CYP4F12, RERE* and *PLAU* seemed to drive separation of the patients in an opposite direction than *AR*. On the contrary, PC1 from MIBC cases, which explained 52.17% of the variance in the data, separates patients mainly on the bases of *AR* levels and shows that *AR, PPP1R3C, CYP4F12* and *RERE* drive this separation in an opposite direction than *PLAU*. All five genes contributed to segregation of the data with loadings greater than 0.3 or lower than −0.3. Similar results were observed in the PCA from the MIBC cases from TCGA (Fig. 6B). Lastly, also in the TCGA group *AR* expression was not significantly different between genders (Fig. 6C). Despite this, the expression of *RERE* was significantly lower in women compared with men, in both our microarray and the TCGA data sets (Fig. 6C). No other significant association was found between *AR, PLAU, PPP1R3C, CYP4F12* or *RERE,* and other clinical features available (data not shown).

## 4 Discussion

AR signalling is one of the main drivers of sex differences and is suggested to be involved in bladder carcinogenesis [9, 37, 38]. Here, we provide evidence that the androgen/AR signalling axis is active and promotes the development of an aggressive phenotype in an UCC‐derived cell line model. Like other UCC‐derived cell lines, the UM‐UC‐3 cell line contains a low percentage of cells that are strongly AR‐positive. Using limited dilution, UM‐UC‐3 cells were subcloned to obtain a fully AR‐positive cell line (UM‐UC‐3‐AR). Based on the *AR* mRNA levels, UM‐UC‐3 and UM‐UC‐3‐AR represent the UCC cases with low‐ and high*‐AR* expression levels, respectively. Both lines UM‐UC‐3 and UM‐UC‐3‐AR contain the same number of glycine repeats within exon 1, the same number of X chromosomes and the same *AR* gene copy number. Thus, additional levels of gene regulation should underlie the different *AR* mRNA expression levels in these two isogenic cell lines, which also could explain the differences in AR levels found in bladder tumours.

Although AR levels in UM‐UC‐3‐AR were significantly lower than those in LNCaP, reflecting difference between UCC and PCa patients, these levels were sufficient to mediate phenotypic changes. Ligand‐bound AR regulated the clonogenic capacity of UM‐UC‐3‐AR cells. Androgen stimulation resulted in the formation of big‐sized holoclones. Addition of AR inhibitor enzalutamide significantly reduced colony formation, and similarly to siRNA‐mediated knockdown, it resulted in the generation of small paraclones. PCa‐derived cells, seeded at low densities, have been shown to form three different colony morphologies *in vitro* that can be classified as holoclones, meroclones and paraclones. These morphologies are suggested to represent stem cells, early progenitor cells and late progenitor cells, respectively [34, 35]. The formation of big colonies from UM‐UC‐3‐AR upon hormonal stimulation reflects the self‐renewal ability of holoclones. Likewise, the formation of colonies from single cells reflects the capacity of cells to grow independent of cell‐to‐cell interactions, also characteristic of cells with increased cell migration capabilities [39]. Together, this suggests that both AR‐mediated effects in cell migration and colony formation in UM‐UC‐3‐AR are tightly linked.

With these phenotypic changes, several genes were significantly upregulated upon hormone stimulation, among which some previously described for their role in promoting migration and colony formation of diverse type of cancer models such as *FKBP5, LDH‐A, GREB1, PDLIM1, TNK2, VASP, ARHGAP31* and *ZHX3* [40, 41, 42, 43, 44, 45, 46, 47], and genes known to drive the development of cancer cells with stem cell‐like characteristics such as *TFCP2L1* and *SH3RF3* [48, 49]. These genes were consistently downregulated upon treatment with enzalutamide or after AR knockdown, which validates them as androgen‐responsive genes.

Androgen treatment of UM‐UC‐3‐AR cells also induced expression of *NNMT* and *LDHA*, genes previously reported to be upregulated in UCC patients [50, 51], and of *BCL6* and *H19*, whose high expression in UCC is associated with an adverse prognosis [52, 53]. Consistently, loss of or reduced expression of *SOCS6* and *SEMA3F*, genes downregulated upon androgen treatment in our model, is associated with a malignant phenotype in UCC [54, 55]. These studies are in agreement with the phenotype observed in our UCC model, and it presents the AR signalling axis as a novel regulatory mechanism for the expression of these genes in UCC.

GSEA demonstrated that transcriptome dynamics observed in UM‐UC‐3‐AR indeed correspond to an androgen response signature, which included genes encoding for the AR co‐regulators PIAS1 and CCND3 [56, 57], and AR‐interacting partners CDK6, NDRG1 and FKPB5 [58, 59, 60]. The hypoxia‐inducible gene *LDH‐A* was also part of the hypoxia signature in our enrichment analysis, the pathway with highest NES in response to androgen stimulation. *LDH‐A* relates to stem cell function and is associated with poor postradiotherapy outcome in UCC [61]. The oestrogen response pathway was also associated with an androgen‐ and AR‐dependent response in our UCC cell line model. Cross‐talk between steroid receptor pathways has been described, and target genes often harbour response elements for multiple steroid receptors [62]. Indeed, the AR target gene *GREB1* was first reported to be oestrogen‐responsive [63] and later shown also to be regulated by androgens through AR‐mediated transcription [64]. Other signatures shown in this study have been linked to UCC development and progression before. Perturbed bile acid biosynthesis [65] and elevated glycolysis [66] have been reported in UCC tumours. The activation of aerobic glycolysis provides energy to cancer cells and supports their anabolic growth. However, further research is needed to understand the exact mechanisms by which AR regulates the expression of genes associated with these pathways.

Four genes *CYP4F12*, *PPP1R3C*, *RERE* and *PLAU* defined as androgen‐responsive in UM‐UC‐3‐AR significantly correlated with *AR* in two independent UCC data sets. The *CYP4F12* gene encodes a member of the cytochrome P450 (or CYP) superfamily of enzymes, which are involved in the synthesis of cholesterol and steroids (e.g. androgens), and in drug metabolism. The latter function being particularly important in tissues regularly exposed to disposal products (e.g. medicine metabolites, carcinogens) such as the bladder. Indeed, deregulation of some CYP proteins has been proposed as a risk factor for the development of bladder cancer [67, 68], but little is known about the role of CYP4F12, in particular, in bladder cancer progression. Interestingly, *CYP4F12* mRNA expression was shown to be downregulated upon treatment with AR inhibitors enzalutamide or ARN‐509 in PCa‐derived cells [69], in line with the results observed in UM‐UC‐3‐AR. Transcript levels of *PPP1R3C*, a gene that encodes a protein involved in glycogen synthesis and part of the ‘hypoxia’ and the ‘myogenesis’ signatures from our GSEA, were also shown before to be androgen‐responsive [70], as opposed to *PLAU,* a gene that is known to be downregulated by androgens in PCa models [71]. Both reports are consistent with the observations in our UCC‐derived cell line. Lastly, upregulation of *RERE* mRNA as a consequence of miR‐22 inhibition resulted in increased migration and cell proliferation in colon cancer [72]. Curiously, downregulation of this tumour suppressor microRNA has been associated with hypermethylation of miR‐22 genomic AR‐binding sites in PCa [73]. Although these reports strengthen the link between *AR* and these four genes, the direct or indirect role of AR protein in the regulation of *CYP4F12, PPP1R3C, RERE* and *PLAU* transcription and function in UCC is yet to be elucidated.

Our transcriptome data also revealed a cluster of androgen‐responsive genes downregulated upon *AR* knockdown that were not affected by enzalutamide treatment. Enzalutamide promotes the accumulation of AR in the cytosol [74], which could potentiate nongenomic (i.e. non‐nuclear) AR signalling. In the cytoplasm, ligand‐bound AR interacts with protein kinases such as SRC and PI3K via its N terminus to initiate signal transduction pathways that modulate cellular proliferation and migration in prostate cancer cell lines. This process has been reported to be unresponsive to AR antagonists such as enzalutamide [36]. The transcriptional response in cluster 2 from our UCC cell line, however, is different than that described for nongenomic AR signalling in PCa, and thus, future studies are required to unravel the cytoplasmic functions of AR in UCC.

A negative association between AR expression and tumour stage has been reported before, both at the mRNA and protein levels [75]. Indeed, transition into muscle‐invasive stages has been proposed to be initially regulated by AR and as the disease advances it acquires an AR‐independent profile [76]. Our patient data showed that *AR* mRNA levels were significantly upregulated in NMIBC compared with MIBC. The lower frequency of AR‐positive cases in MIBC also suggests that AR‐independent but androgen‐dependent mechanisms support high stage disease. Certainly, a recent publication has shown that the SLC39A9 membrane protein acts as an AR that, when activated by androgens, increases migration and invasion of AR‐negative MIBC cells *in vitro* and *in vivo* [77]. Our transcriptome data also revealed a cluster of genes being regulated by androgens in an AR‐independent manner. Thus, ADT directed to lower systemic androgen levels may be effective in most MIBC cases.

Several studies, including ours, reported that the incidence of UCC is three to four times higher in men than in women [5, 6]. However, discrepancies exist in the number of AR‐positive UCC cases [75, 78], which could be explained by differences in patient populations and detection methods used in different studies. All of our bladder specimens expressed *AR* mRNA levels, both in the microarray analysis and in the RT‐qPCR validation. A subset of UCC tumours, both NMIBC and MIBC, expressed *AR* mRNA levels above those detected in nonmalignant bladder urothelium specimens. Our *in vitro* data indicate that androgen/AR signalling is involved in the acquisition of a more aggressive phenotype of bladder tumour cells and that it can be reverted with blockage of AR signalling, advocating the potential benefits of AR‐targeted therapy for UCC. In low‐grade NMIBC, AR‐targeted therapy may prevent the recurrence of disease, and in high‐grade NMIBC and MIBC, it may prevent progression. Indeed, in PCa patients with concomitant NMIBC, progression of NMIBC to MIBC was only reported in patients who did not receive ADT as a treatment for their prostate tumour [11]. Furthermore, our correlation data suggest a relevance for AR signalling at late stage of UCC. This means ADT could be relevant to those MIBC described in our study, which expressed high‐*AR* mRNA levels. Together, our data provide new understandings in the role of androgens and AR signalling in UCC and suggest that ADT is a realistic option for the treatment of this disease.

## 5 Conclusions

Our study provides biological evidence of AR signalling in UCC with several pathways being differentially regulated in response to androgen treatment. Ligand‐bound AR activates canonical and noncanonical AR target genes and potentiates migration and colony formation. The contribution of AR to the development of an aggressive phenotype in UCC highlights its potential clinical relevance as a novel therapeutic target.

## Conflict of interest

The authors declare no conflict of interest.

## Author contributions

MVLV, GWV and JAS contributed to the experimental design. MVLV, JJD, MA‐H, FS and GvZ performed experiments and data analysis. MVLV prepared the manuscript. GWV, JJD, JPMS, MA‐H, FS, GvZ, DS, MV and JAS revised the manuscript.

### Peer Review

The peer review history for this article is available at https://publons.com/publon/10.1002/1878‐0261.12957.

## Supporting information


**Fig. S1**. *AR* expression in bladder urothelial cell carcinoma tissue samples.

**Fig. S2**. Characterization of UM‐UC‐3 and UM‐UC‐3‐AR.

**Fig. S3**. Colony formation assay.

**Fig. S4**. Migration capacity of UM‐UC‐3 cells.

**Fig. S5**. Expression of androgen‐responsive genes and AR in UCC patients.

**Table S1**. Clinical information of bladder urothelial cell carcinoma patients from the Nijmegen cohort used in microarray analysis.
**Table S2**. Clinical information from bladder urothelial cell carcinoma patients used in RT‐qPCR analysis.
**Table S3**. Primer sequences for real‐time semi‐quantitative (RT)‐qPCR analysis.
**Table S4**. Primer sequences for glycine repeat and gene copy number analysis.
**Table S5**. siRNA sequences.
**Table S6**. Transcript expression data from muscle‐invasive bladder cancer cases from the TCGA database.
**Table S7**. Clinical information of muscle invasive bladder cancer patients, from publically available TCGA used for *in silico* analysis.
**Table S8**. RT‐qPCR Cp values from bladder urothelial cell carcinoma and non‐malignant urothelium tissue samples.
**Table S9**. Transcriptome profile after Enzalutamide treatment or siRNA‐mediated AR knockdown of androgen‐treated UM‐UC‐3‐AR cells.
**Table S10**. Microarray expression values of AR and 265 androgen‐AR responsive genes (identified in UM‐UC‐3‐AR) in 97 bladder urothelial cell carcinoma patients from the Nijmegen cohort.
**Table S11**. Median expression (log_2_) of AR and 266 androgen‐responsive genes (identified in UM‐UC‐3‐AR) in 97 bladder urothelial cell carcinoma (UCC) patients from the Nijmegen cohort (microarray data set).
**Table S12**. Spearman correlation coefficient from subset of androgen‐responsive genes correlating with AR in bladder urothelial cell carcinoma patients from the Nijmegen cohort (microarray data set).
**Table S13**. Median expression (log_2_) of AR and 254 androgen‐responsive genes (identified in UM‐UC‐3‐AR) in 408 muscle invasive bladder cancer patients, from the TCGA cohort.
**Table S14**. Spearman correlation coefficient from subset of androgen‐responsive genes correlating with AR in MIBC patients from TCGA.

## Data Availability

The data that support the findings of this study are available in the [Link], [Link], [Link], [Link], [Link], [Link] of this article.
