# Supplementary figures and images for: Androgen receptor signalling confers clonogenic and migratory advantages in urothelial cell carcinoma of the bladder
Source: Mol Oncol. 2021 May 22;15(7):1882–900. doi: 10.1002/1878-0261.12957 (PMC8253097; doi:10.1002/1878-0261.12957)

**A**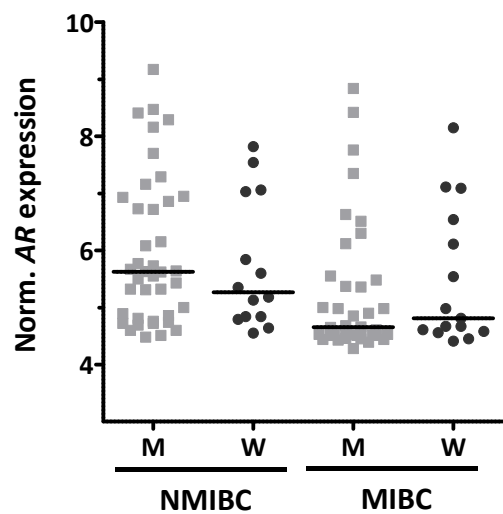**B**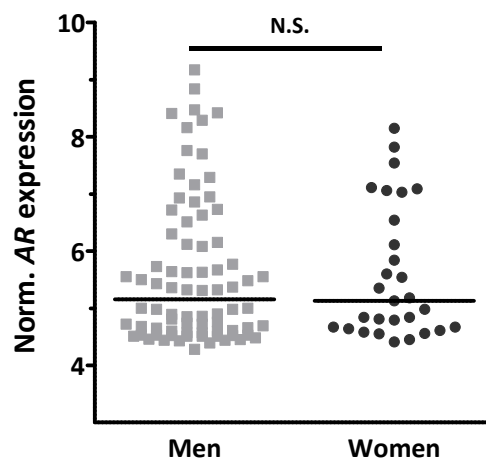**C**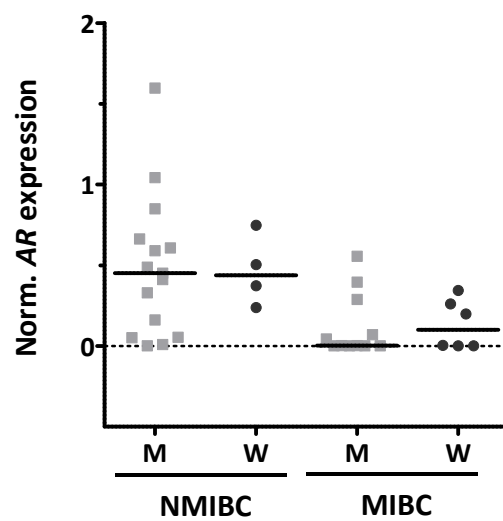**D**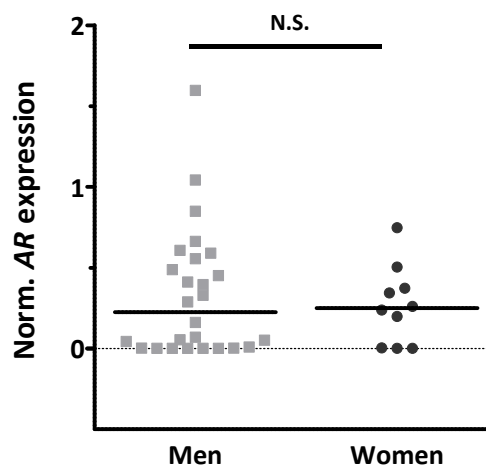

Supplement: Supplementary file 1 — Fig. S1. AR expression in bladder urothelial cell carcinoma tissue samples. [file MOL2-15-1882-s006.pdf]

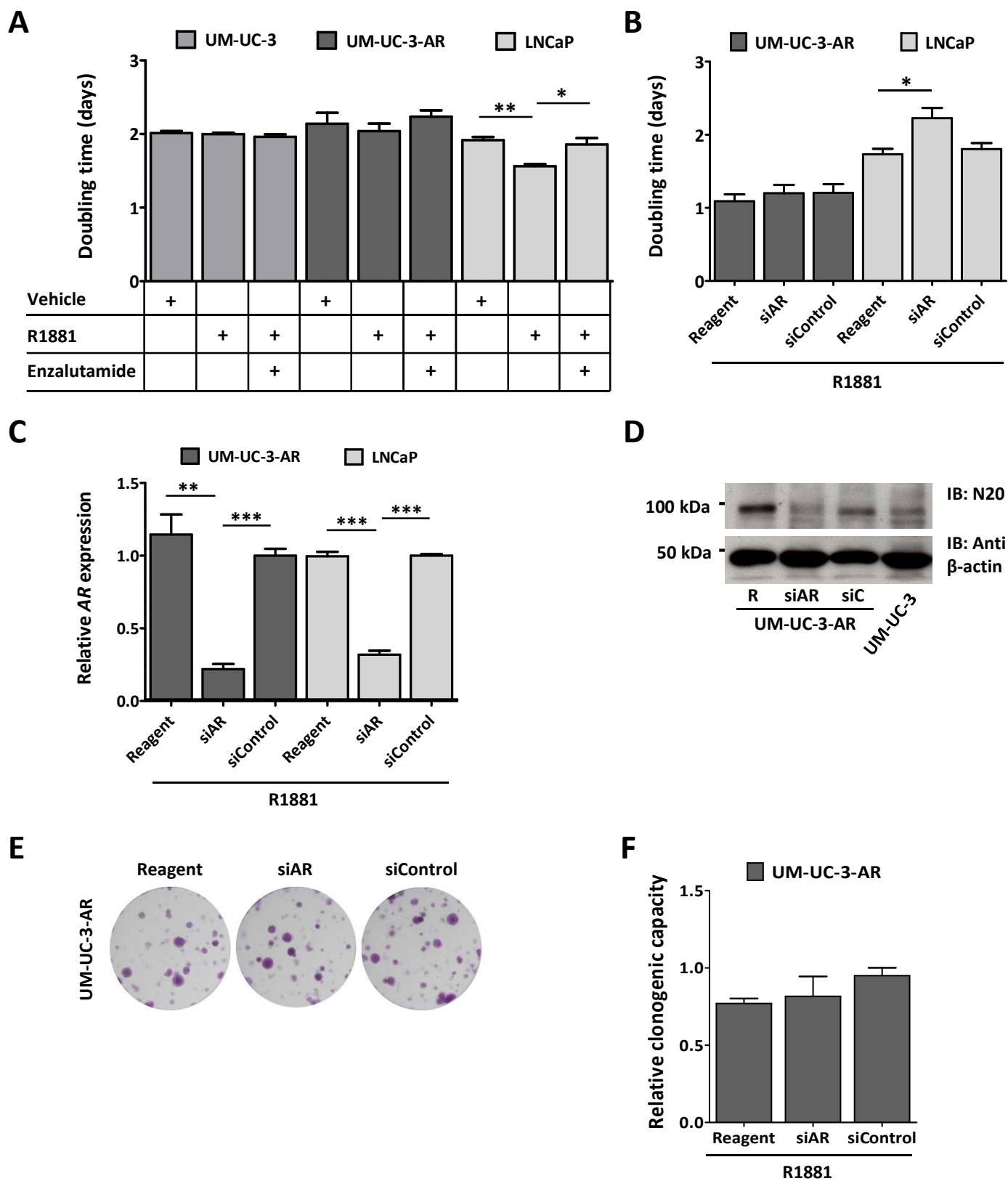

Supplement: Supplementary file 3 — Fig. S3. Colony formation assay. [file MOL2-15-1882-s001.pdf]

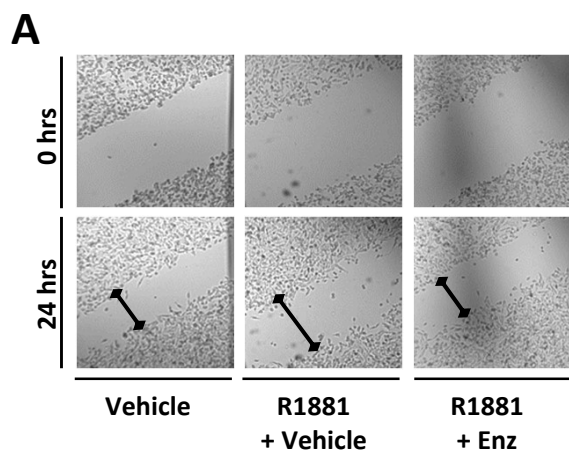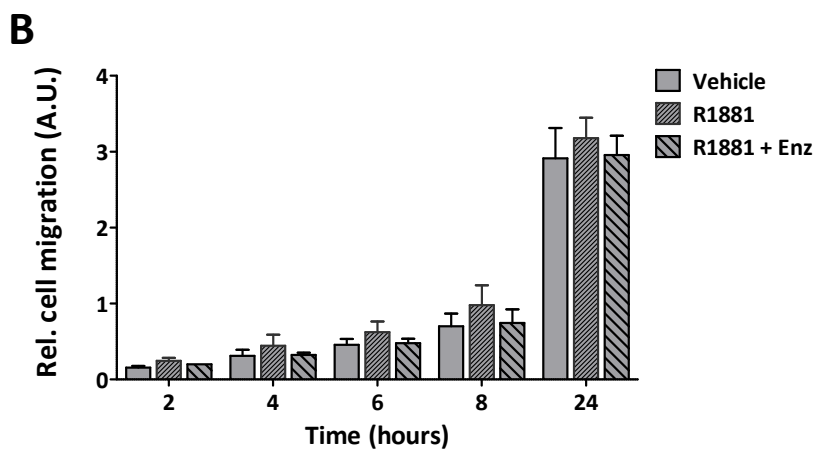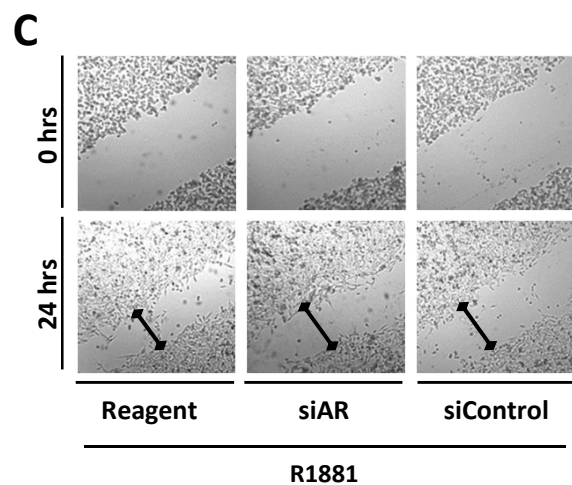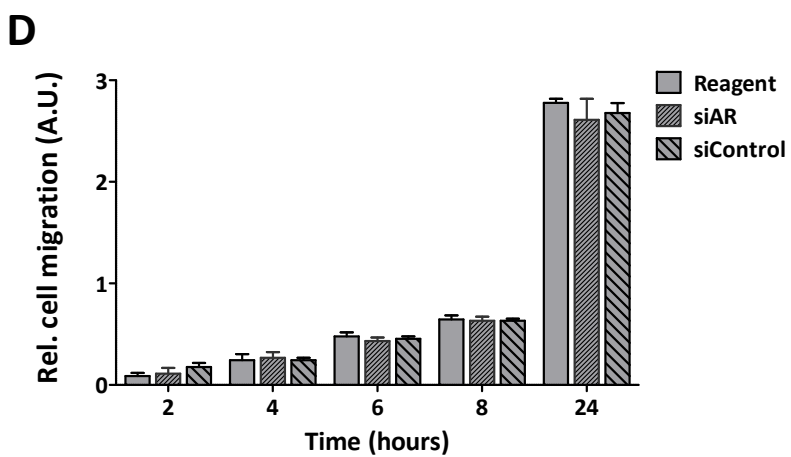

Supplement: Supplementary file 4 — Fig. S4. Migration capacity of UM‐UC‐3 cells. [file MOL2-15-1882-s002.pdf]

# A

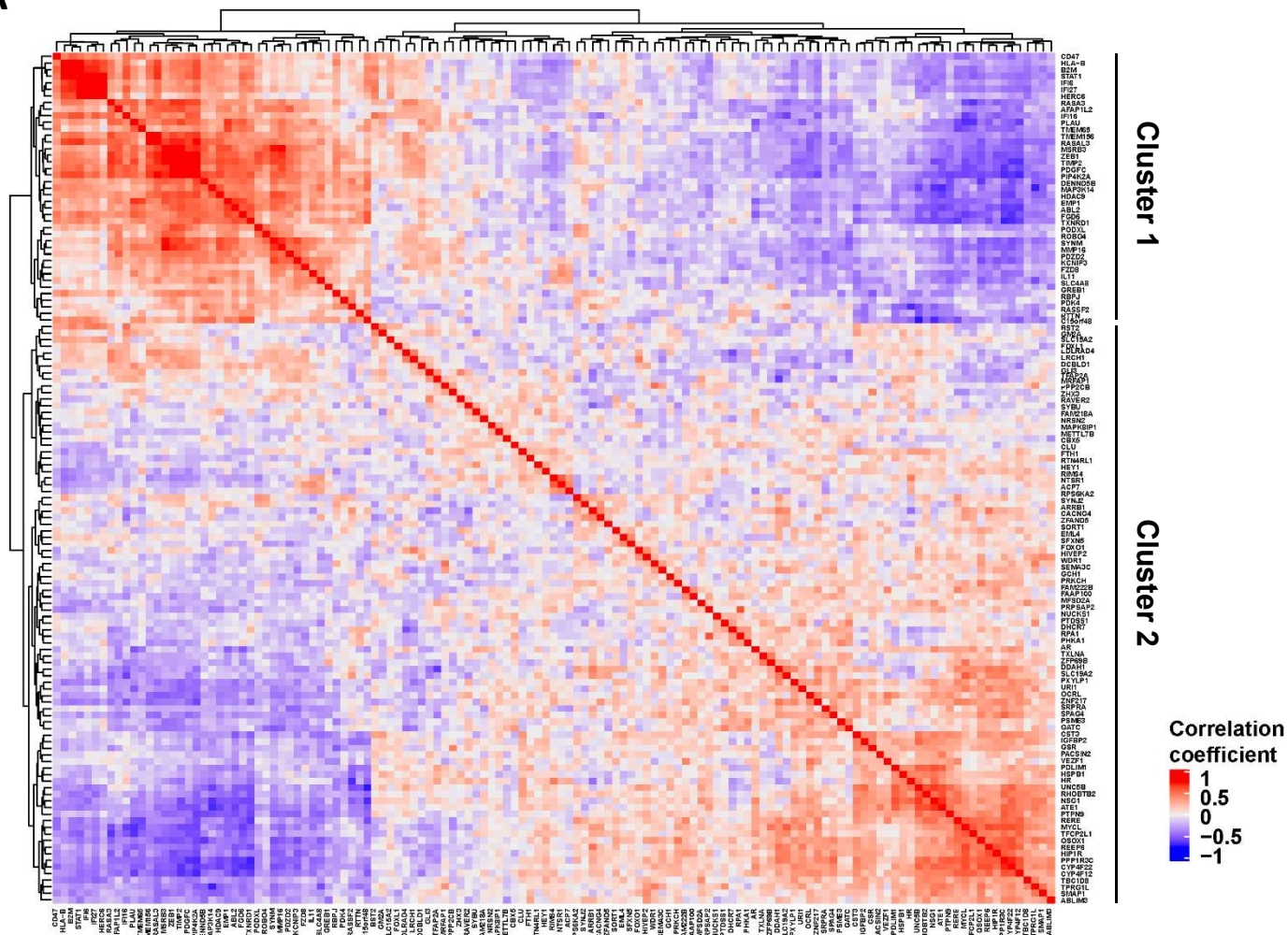

# B

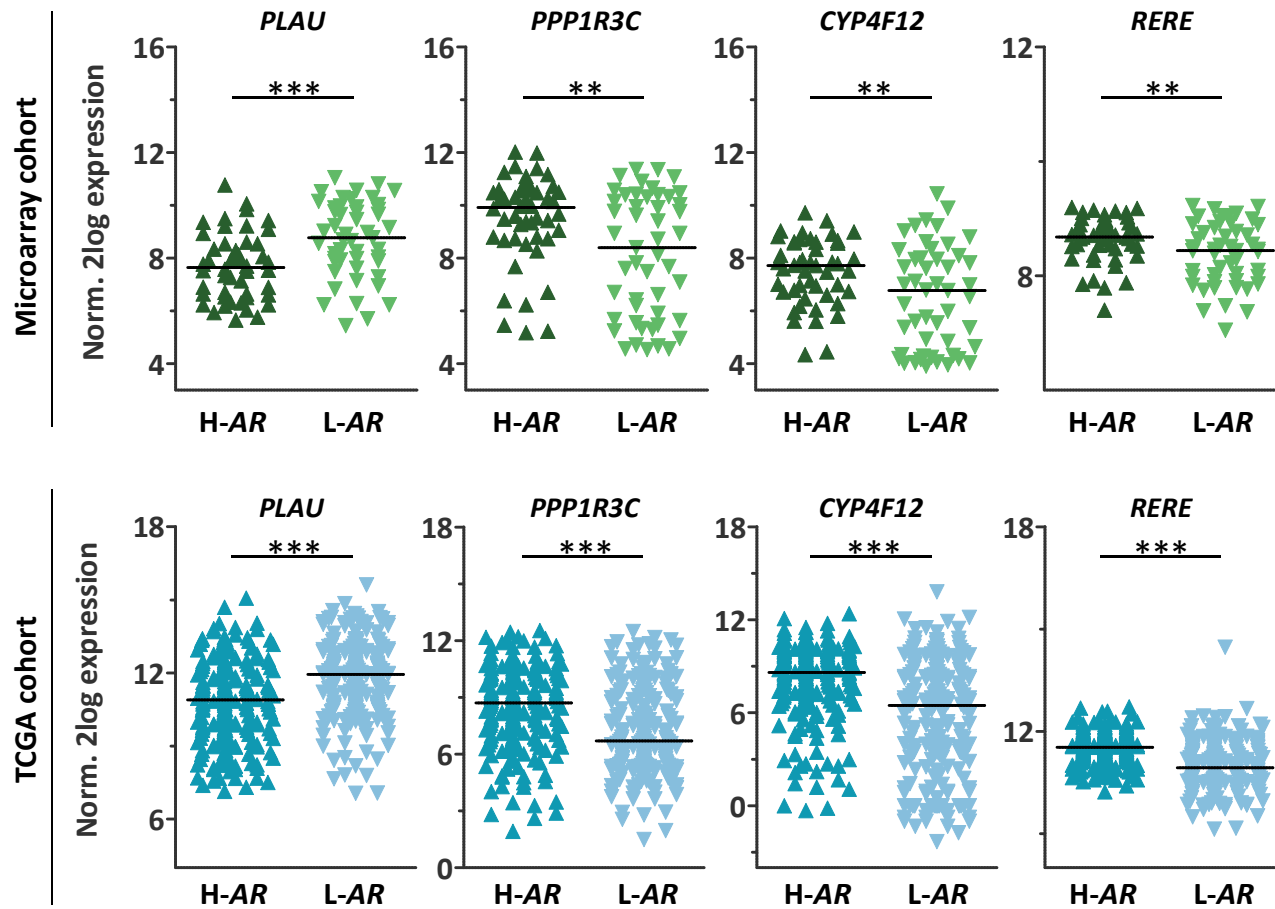

Supplement: Supplementary file 5 — Fig. S5. Expression of androgen‐responsive genes and AR in UCC patients. [file MOL2-15-1882-s003.pdf]
