# Supplementary material for: Androgen receptor signalling confers clonogenic and migratory advantages in urothelial cell carcinoma of the bladder
Source: Mol Oncol. 2021 May 22;15(7):1882–900. doi: 10.1002/1878-0261.12957 (PMC8253097; doi:10.1002/1878-0261.12957)
Supplement: Supplementary file 2 — Fig. S2. Characterization of UM‐UC‐3 and UM‐UC‐3‐AR. [file MOL2-15-1882-s004.pdf]

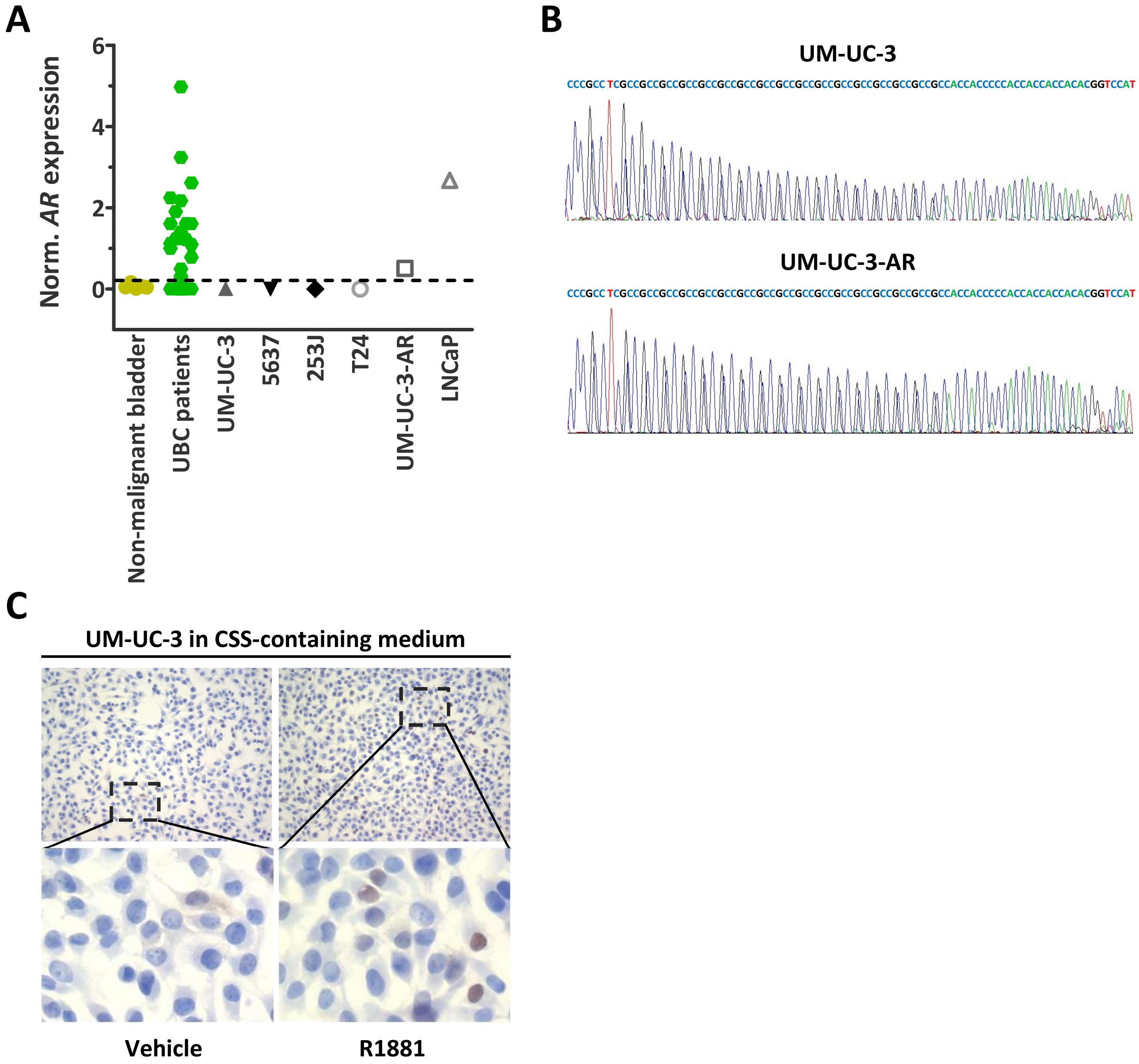
A
Norm. AR expression
6
4
2
0
Non-malignant bladder
UBC patients
UM-UC-3
5637
253J
T24
UM-UC-3-AR
LNCaP
B
UM-UC-3
CCCGCC TCGCCGCCGCCGCCGCCGCCGCCGCCGCCGCCGCCGCCGCCGCCGCCGCCGCCACCACCCCCACCACCACCACACGGTCCAT
UM-UC-3-AR
CCCGCC TCGCCGCCGCCGCCGCCGCCGCCGCCGCCGCCGCCGCCGCCGCCGCCGCCGCCACCACCCCCACCACCACCACACGGTCCAT
C
UM-UC-3 in CSS-containing medium
Vehicle
R1881
